# A Simple Method to Manufacture a Force Sensor Array Based on a Single-Material 3D-Printed Piezoresistive Foam and Metal Coating

**DOI:** 10.3390/s24123854

**Published:** 2024-06-14

**Authors:** Claude Humbert, Mathis Barriol, Sakine Deniz Varsavas, Pascal Nicolay, Mathias Brandstötter

**Affiliations:** 1 CiSMAT—Carinthia Institute for Smart Materials, Carinthia University of Applied Sciences, 9524 Villach, Austria; 2 ADMiRE—Additive Manufacturing, Intelligent Robotics and Engineering, Carinthia University of Applied Sciences, 9524 Villach, Austria

**Keywords:** sensor array, 3D printing, metal coating, lattice structure, mono-material, piezoresistive foam

## Abstract

Nowadays, 3D printing is becoming an increasingly common option for the manufacturing of sensors, primarily due to its capacity to produce intricate geometric shapes. However, a significant challenge persists in integrating multiple materials during printing, for various reasons. In this study, we propose a straightforward approach that combines 3D printing with metal coating to create an array of resistive force sensors from a single material. The core concept involves printing a sensing element using a conductive material and subsequently separating it into distinct parts using metal-coated lines connected to the electrical ground. This post-printing separation process involves manual intervention utilizing a stencil and metallic spray. The primary obstacle lies in establishing a sufficient contact surface between the sprayed metal and the structure, to ensure effective isolation among different zones. To address this challenge, we suggest employing a lattice structure to augment the contact surface area. Through experimental validation, we demonstrate the feasibility of fabricating two sensing elements from a single-material 3D-printed structure, with a maximum electrical isolation ratio between the sensors of above 30. These findings hold promise for the development of a new generation of low-tech 3D-printed force/displacement sensor arrays.

## 1. Introduction

Force sensors and strain gauges are extensively utilized in various applications across many fields. These sensors play a crucial role in monitoring structures for civil engineering applications [1,2,3,4,5,6,7,8] and various machinery in different domains of engineering, contributing to improved performance and safety [9,10,11]. Additionally, the versatility and precision of these sensors have attracted interest in the medical sector, where they find application in biomechanics, patient monitoring, and medical device development [12,13,14,15,16,17,18,19,20,21,22,23,24,25].

The introduction of 3D printing has not only revolutionized sensors’ customizability [26,27,28,29,30,31,32] but has also facilitated the creation of flexible and wearable force and strain sensors that are more suitable for medical applications [33,34,35,36,37,38,39,40,41,42,43]. Through 3D printing, these sensors can be integrated into mechanical structures, making them inherently tailorable. This innovative approach allows for the incorporation of sensors within complex mechanical systems, enhancing their functionality. There are three primary methods of integrating sensors into 3D-printed structures [44].

1. Integration of pre-existing sensors into a 3D-printed structure: This method presents notable benefits, such as the utilization of reliable, calibrated, and readily available sensors. Nonetheless, this approach necessitates the adaptation of each design to accommodate the sensor and may even require pausing the printing process to position them, thereby limiting productivity.
2. Integration of 3D-printed sensors in a 3D-printed structure: This approach presents the advantage of crafting customized sensors that match specific design requirements [45]. However, similarly to the first method, it involves one fabrication process per design and multiple fabrication steps.
3. Fully 3D-printed: This method allows for complete tailoring, offering maximum design flexibility. The sensors can be intricately integrated into the mechanical structure, especially with multimaterial printing [46,47], enhancing the overall customization. The challenge lies in ensuring robust electrical connections (poor connections can impact the overall performance of the integrated sensors). Indeed, metals and polymers cannot be printed together, and conductive polymers often show limitations: their electrical resistivity makes them relatively poor candidates for electrodes, and their mechanical fragility can break the connections.

A unified system, where both the structure and sensors are 3D-printed using a single material, stands out as a favorable choice toward customizability.

- By using a single material, the risk of failure due to multimaterial interactions is significantly reduced. This is particularly crucial in applications where electrical connections within the structure are involved.
- This approach eliminates the complexities associated with managing multiple materials during fabrication, resulting in faster and cheaper production cycles.
- The reduced complexity in material usage aligns with sustainable practices. A mono-material system simplifies the recycling processes, contributing to a more environmentally friendly approach to manufacturing.

Single-material force sensors can be crafted from a resistive material that undergoes deformation under pressure. In this work, our purpose is to design, fabricate, and test a new type of customizable array of single-material and 3D-printed resistive force sensors. The main question to address is how to design multiple sensors out of a single structure. Our solution is to combine 3D printing with metal spray coating. Spray coating has previously been employed in conjunction with 3D printing to produce conductive polymer components or to directly create electrodes on printed parts [48,49]. Nevertheless, these processes necessitate costly equipment and meticulous design considerations. The alternative approach presented here is centered on post-print spray coating. This allows for the utilization of standard printing processes and conventional printers, which enlarges the potential target group of 3D-printed polymer structures. The simplicity and cost-effectiveness of this method are enhanced by employing a basic stencil and manual spray coating. The conductivity of the spray coating is substantial, and its connection to the electrical ground makes possible the segmentation of the structure into distinct sections, enabling multiple sensing within a single structure.

However, a significant challenge lies in establishing effective surface contact between the structure and the coating to ensure proper shielding. Our solution relies on a lattice structure geometry, which not only has the potential to addresses this contact surface challenge but also provides the added benefit of adjusting the stiffness of the structure and amplifying its sensitivity to electrical resistance changes in response to the applied forces. Such softer, force-sensitive structures are known as piezoresistive foams. Piezoresistive foams have attracted interest in the research community for several years [50,51,52,53,54,55,56]. They offer a wide range of potential applications as they can be used to create pressure sensors.

- Smart Textiles: The integration of such foams into fabrics can create smart textiles capable of sensing pressure or deformation, enabling applications like interactive clothing, posture-monitoring systems, or healthcare garments.
- Biomedical Devices: In biomedical engineering, piezoresistive foams can find application in pressure-sensitive implants or prosthetic limbs to provide feedback about the pressure distribution or movement.
- Robotics: These foams can be utilized in robotic systems for tactile sensing, allowing robots to perceive and respond to the forces applied to their surfaces. This can enhance their ability to interact safely and effectively with the environment.
- Sports Equipment: Incorporating piezoresistive foams into sports equipment such as helmets, pads, or shoes can provide real-time feedback on impact forces, helping athletes to monitor and prevent injuries.

The ultimate design presented in this paper incorporates two piezoresistive foam sensing elements within a single structure. However, it is crucial to note that this work does not seek to fully develop a new high-performing and calibrated sensor. Instead, its primary objective is to serve as a demonstration, showcasing the potential and feasibility of the proposed conceptual fabrication process. The selected geometry and its printing, coating process, and electronic readout will first be presented. Then, the testing conditions will be described, followed by the presentation of both simulations and experimental results. Finally, an analysis of these results will be provided to draw conclusions and insights into the effectiveness of the method.

## 2. Materials and Methods

**Figure 1:**
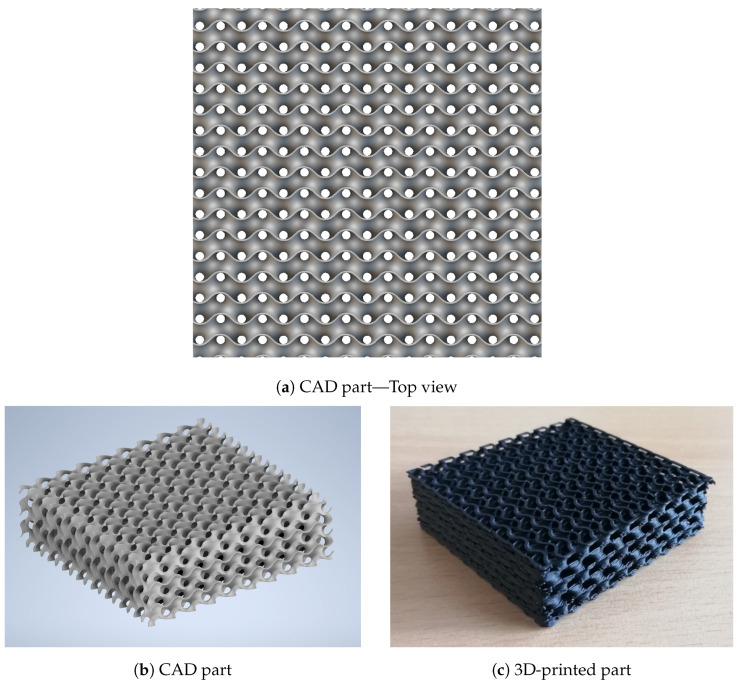
Piezoresistive foam (gyroid lattice structure) developed, studied, 3D-printed, and tested in this work. It is referred as the “structure”.

In order to illustrate the concept of electrically splitting a single-material conductive structure, we devised a cubic structure measuring 5cm × 5cm × 1.5cm, crafted through 3D printing and shown in Figure 1.

This structure is made of a resistive thermoplastic (conductive filaflex from Recreus Industries). This material has been chosen for its low resistivity (3.9 Ωcm) and high elongation at break (above 100%). The printed pattern is a Gyroid filled at 15% with a wavelength of 6mm. We have empirically selected a wavelength that is large enough to enable the 3D printing of the geometry and yet sufficiently small to effectively mimic the mechanical structure of a foam at a macroscopic scale. This lattice structure serves as both the physical framework and the sensing element. This geometry deforms under an applied force, causing more contact points to be created within the lattice structure and therefore inducing a reduction in its electrical resistance, and this piezoresistive effect has the potential to lead to high sensitivity due to these local phenomena.

**Figure 2:**
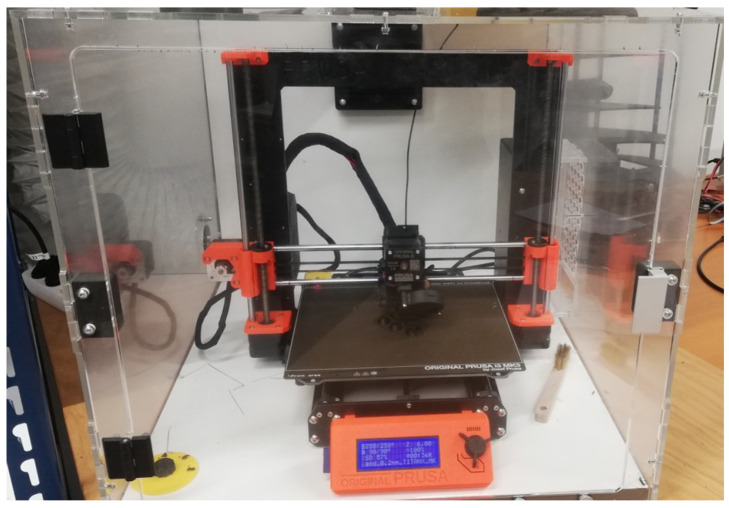
The 3D printer used to fabricate the piezoresistive foam (structure) with the conductive material: Original Prusa i3 MK3S+.

Finally, the printer used, depicted in Figure 2, is the Original Prusa i3 MK3S+. The layer height is 0.2mm, the printing speed is 80mm/s, the nozzle temperature 250 °C, and the bed temperature 50 °C. The selected lattice structure, being one of the default patterns of this printer, is directly generated by the printer to allow a higher likelihood of success during the printing process.

The innovative approach proposed in this article entails partitioning this 3D-printed lattice structure, referred to as the “structure”, into two distinct sensing zones, each termed a “sensor”. This division is achieved by the post-printing application of a metallic coated line. The chosen spray is a copper–iron mixture that has been selected for its relatively low surface resistivity of approximately $0.5\;\Omega /m^{2}$ at a thickness of 50 μm and its reasonable price. A stencil is first fashioned from tape to delineate a line at the center of the structure, on both sides. The application of the spray is then carried out manually. Once dry, this coated line is electrically grounded using a copper wire wrapping the structure. This electrode is hereby called the “sprayed ground”.

**Figure 3:**
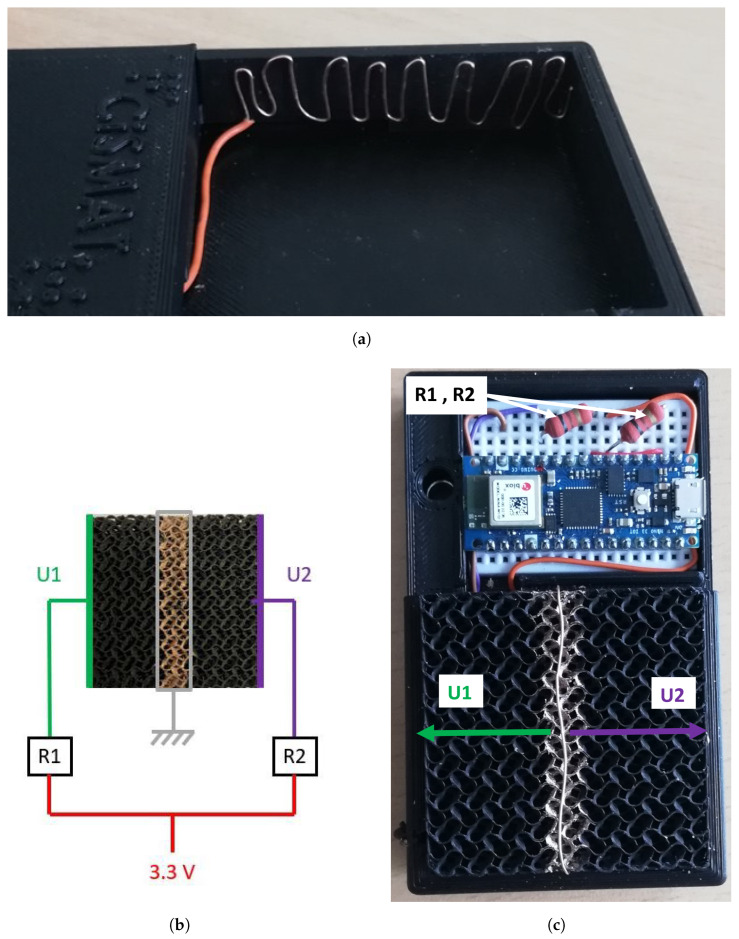
Electrical setup: Arduino Nano, prototype board, resistances $R_{1}$ and $R_{2}$, sensor electrodes, sprayed ground, frame, and structure. (**a**) A sensor electrode positioned in the frame (without the structure). (**b**) Schematics of the connections between the structure and the Arduino. The grey wire is connected to the ground. (**c**) Picture of the structure and its electronics in the frame.

We have utilized black PLA to 3D-print the housing, referred to as the “frame” in this paper, intended to position both the structure and the electronics described below. Thanks to its sprayed ground, the structure is divided into two sensors, numbered 1 and 2. The voltages across them are named $U_{1}$ and $U_{2}$, respectively. These voltages are measured by means of two electrodes, called “sensor electrodes”, which are also fashioned from copper wires and feature a zigzag shape to enhance the surface contact, as depicted in Figure 3a. They are positioned on the sides of the structure, squeezed between the structure and the frame to ensure good surface contact. Each of them is connected with resistance of 100 Ω ($R_{1}$ and $R_{2}$) to establish two voltage dividers, as illustrated in Figure 3b. This resistance value has been empirically chosen to align with the unloaded sensor’s resistance, ensuring the attainment of the highest voltage output sensitivity. Both voltage dividers are interfaced with an Arduino Nano, as shown in Figure 3c, set up to provide a 3.3 V input to both resistances and monitor the voltage of each sensor.

## 3. Results

**Figure 4:**
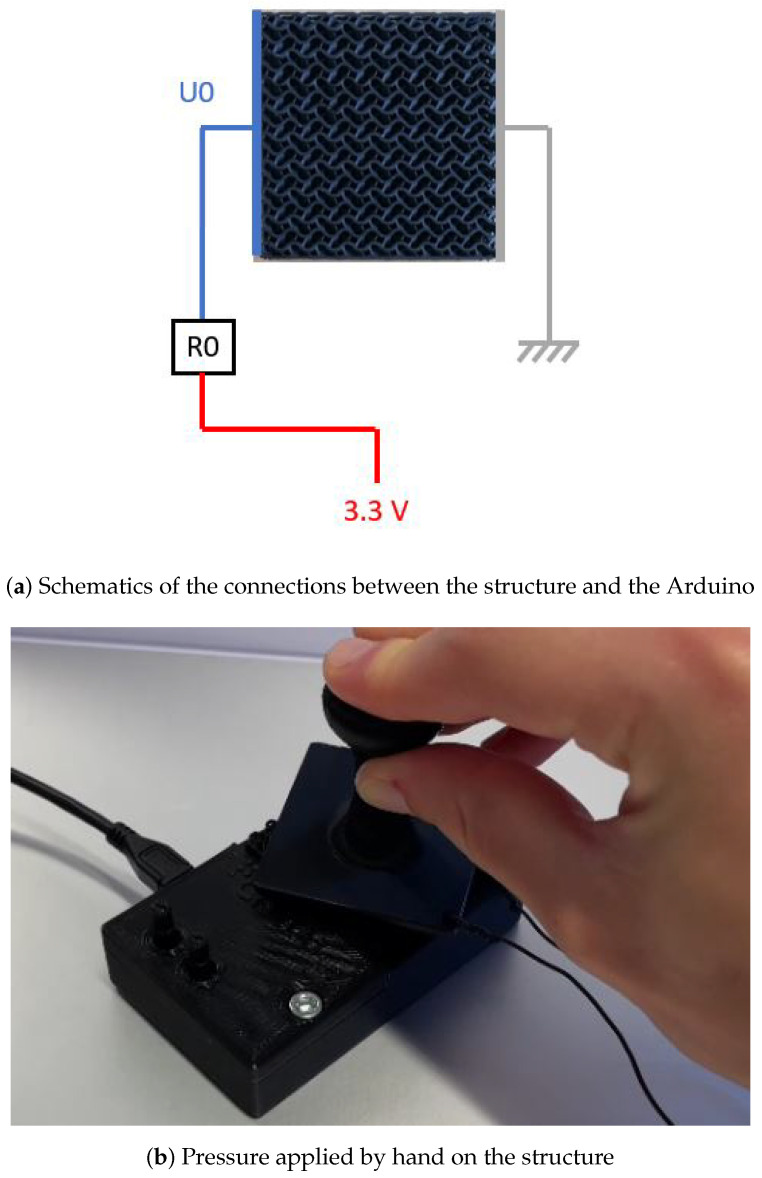

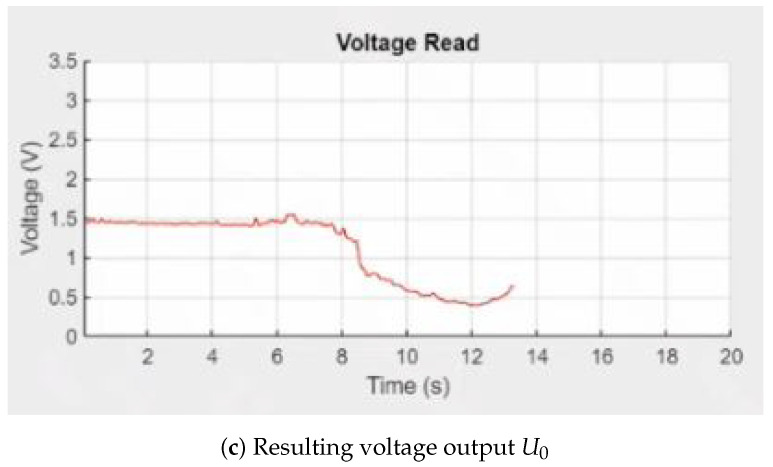
Demonstration of the structure’s piezoresistivity.

**Figure 5:**
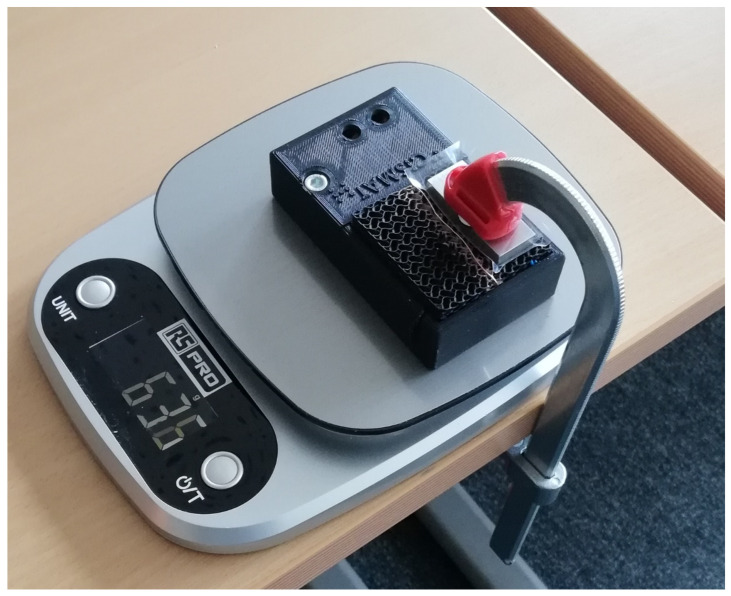
Test setup: structure in its frame connected to its electronics. It is positioned on a scale to measure the force applied by the clamp.

Before conducting experiments on the structure with the sprayed ground, the piezoresistive effect of the structure without the sprayed ground is demonstrated using a similar setup, also based on a voltage divider connected to an Arduino Nano: a single voltage divider is set up, made of a resistor $R_{0}=330\;\Omega$ connected to the first sensor electrode. The second sensor electrode, still positioned on the other side of the structure, is grounded. A force is then applied by hand using a 3D-printed PLA stamp, and the structure voltage is recorded, as depicted in Figure 4. It can be observed that the voltage drops by a factor of 3 with a force applied by hand; it is thereby considered to be qualitatively sensitive enough to validate the positive effect of the selected geometric patterned on the piezoresistivity of the structure. The piezoresistive effect of the structure being validated, the structure with the sprayed ground, is then connected, as described in the previous section. The entire system is positioned on a scale capable of measuring up to 10 kg (approximately 100 N), and the forces are individually applied to one sensor at a time using a clamp (10 steps of 10 N), as illustrated in Figure 5. The primary aim in this configuration is to show that exerting a force on either the left or right half of the structure induces a resistance drop on the same side, leaving the opposite one unaffected.

**Figure 6:**
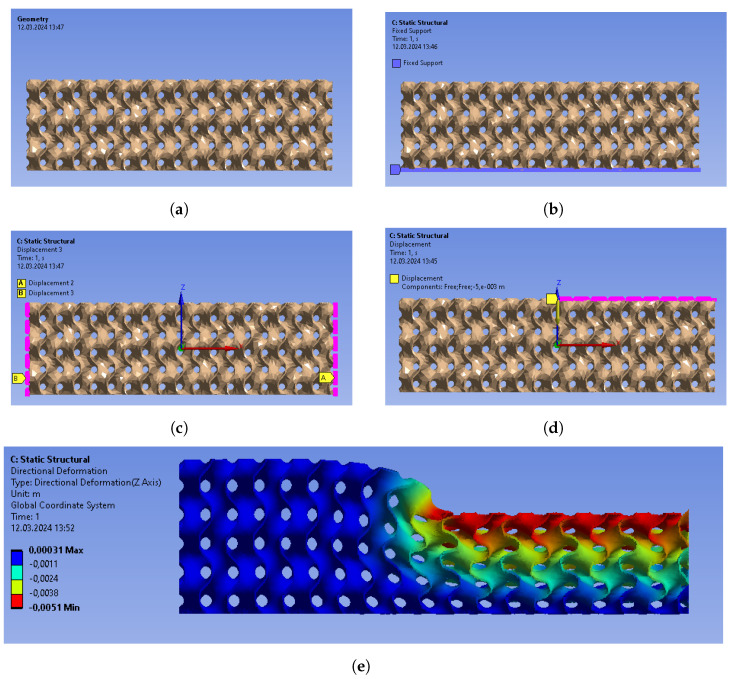
Finite element method simulations (ANSYS): boundary conditions and resulting displacement. (**a**) Geometry. (**b**) Fixed support (in blue). (**c**) Displacement forbidden along the X axis (in pink). The same condition is applied on the two other edges for the Y axis. (**d**) Applied negative displacement along the Z axis (in pink). (**e**) Result: displacement along the Z axis, forced to be 5 mm on the right side and slightly positive on the left side due to the Poisson effect.

In order to illustrate the mechanical response of the structure under a load applied on one of its halves only, a finite element model has been created using ANSYS. In this model, displacement of 5 mm is imposed on the right half of the structure’s top, while its bottom remains fixed. One should note that during the subsequent experiments, a vertical displacement is imposed by the clamp on one half of the structure, while only the resultant force is measured by the scale. In this model, this imposed displacement value of 5mm has only be chosen to illustrate the shape of the structure under our experimental boundary conditions, for both its left and right halves. Additional displacement boundary conditions are applied to this ANSYS model, namely on the sides of the structure, to prevent it from extending beyond the frame (in order to fit the experimental setup). Consequently, the structure becomes thinner on the right side, which should result in straighter paths for electrical currents and thus reduced electrical resistance in this region. Conversely, the left half of the structure experiences slight thickening due to the Poisson effect, leading to a potential increase in its electrical resistance (a classical value of the Poisson ratio has here been used: 0.3). Details regarding the structure’s geometry, the applied boundary conditions, and the simulation results are presented in Figure 6.

**Figure 7:**
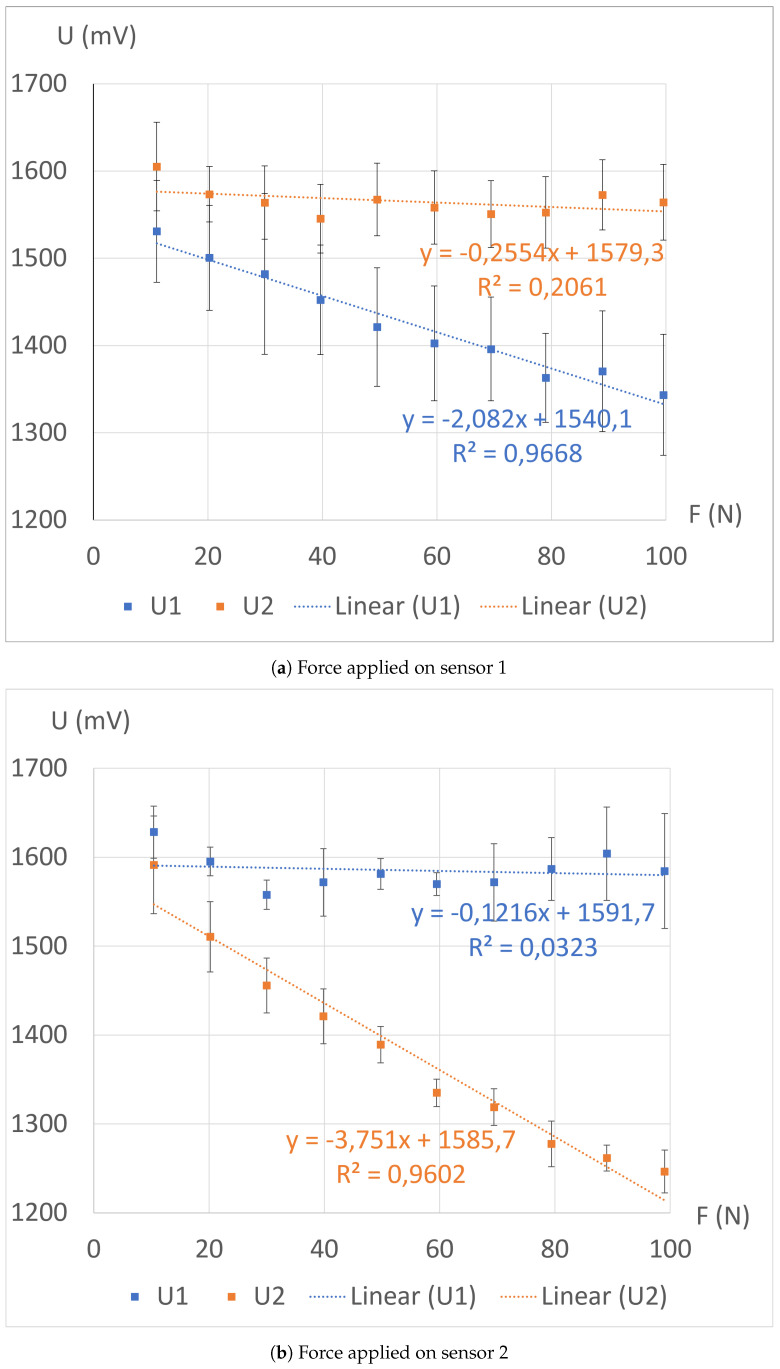
Measured tensions during loading with the clamp, along with their corresponding linear regressions and standard errors.

The experiments described in the previous section have been conducted and the recorded output voltages $U_{1}$ and $U_{2}$ are depicted in Figure 7. The same experiment has been conducted three times in a row on three distinct structures and the voltage has been averaged over the three datasets. It is observed that the voltages of the compressed sensors decrease while they experience an increasing applied force, aligning with our expectations. Conversely, the tensions of the other sensors remain relatively constant. To quantify the sensitivity of each sensor to the applied forces, a linear regression is performed on each dataset, as shown in Figure 7. The slopes obtained from these approximations allow us to perform a first evaluation of the electrical isolation performance of the two sensors. Let $S_{i,j},i,j\in \left\{1,2\right\}$ represent the voltage sensitivity of sensor *i*, with respect to the force *F* applied on sensor *j*. The parameter *S* is thus defined as in Equation (1).

(1) $$S_{i,j}=\frac{dU_{i}}{dF_{j}}$$

The retained isolation performance criterion, denoted $P_{i},i,k\in \left\{1,2\right\},i\neq k$, is then defined as in Equation (2). The interpretation of the value of *P* is as follows. If P=1, it is considered that there is no isolation between the two sensors. If P>1, it can be acknowledged that the metallic spray has indeed an electrical isolation effect.

(2) $$P_{i}=\left|\frac{S_{i,i}}{S_{k,i}}\right|$$

The values of *S* (in mV/N) and *P* extracted from the experimental data are given in Equation (3) and Equation (4), respectively.

(3) $$S=\left[\begin{array}{cc} -2.08 & -0.12\\ -0.26 & -3.75 \end{array}\right]$$

(4) $$P=\left[\begin{array}{c} 8.00\\ 31.2 \end{array}\right]$$

**Figure 8:**
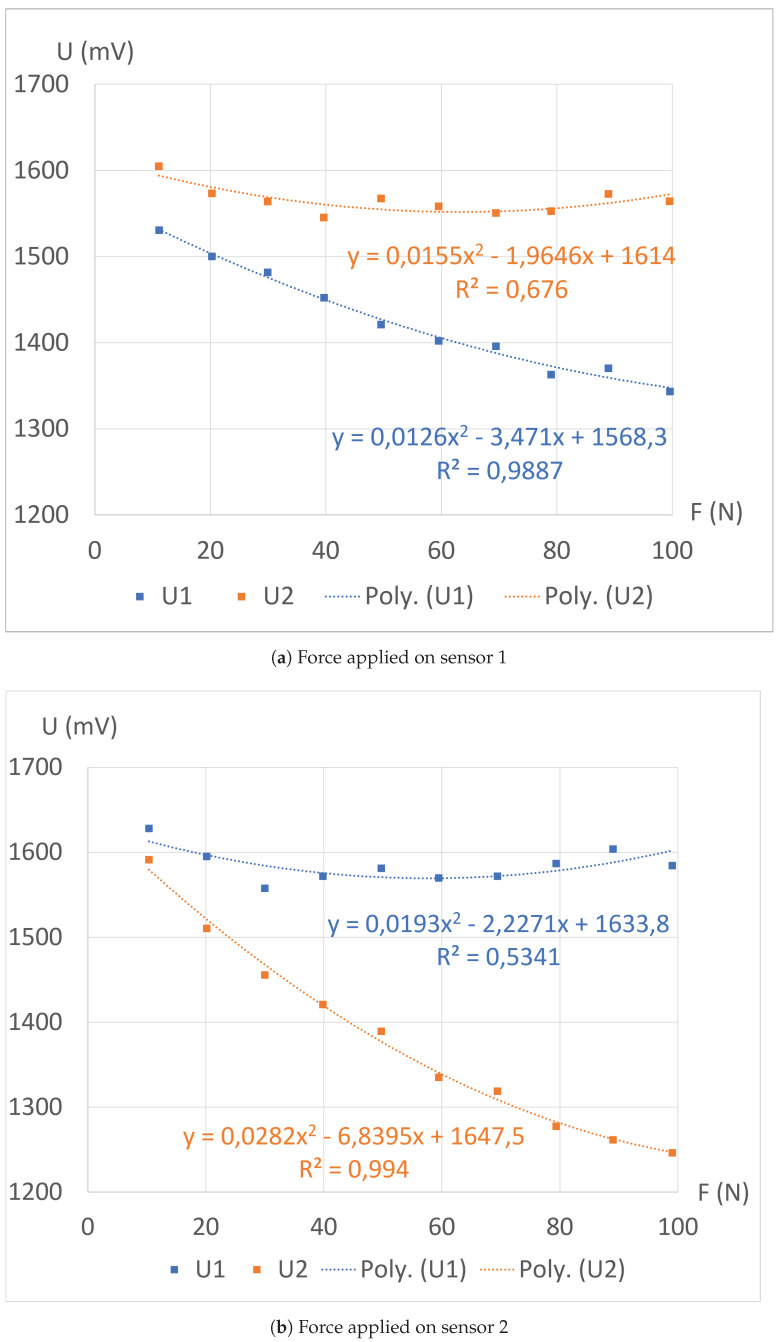
Measured tensions during loading with the clamp, along with their corresponding second-order polynomial regressions.

The values of *P* are greater than 1 and demonstrate the efficacy of the electrical shielding between the two halves of the structure: the sensitivity of the sensors under a mechanical load is at least eight times higher than that of the sensors without any applied mechanical constraint. However, these values are derived from linear regressions with suboptimal coefficients of determination *R* (Figure 7). Consequently, we conduct second-order polynomial regressions on the same datasets, resulting in improved accuracy, as depicted in Figure 8.

**Figure 9:**
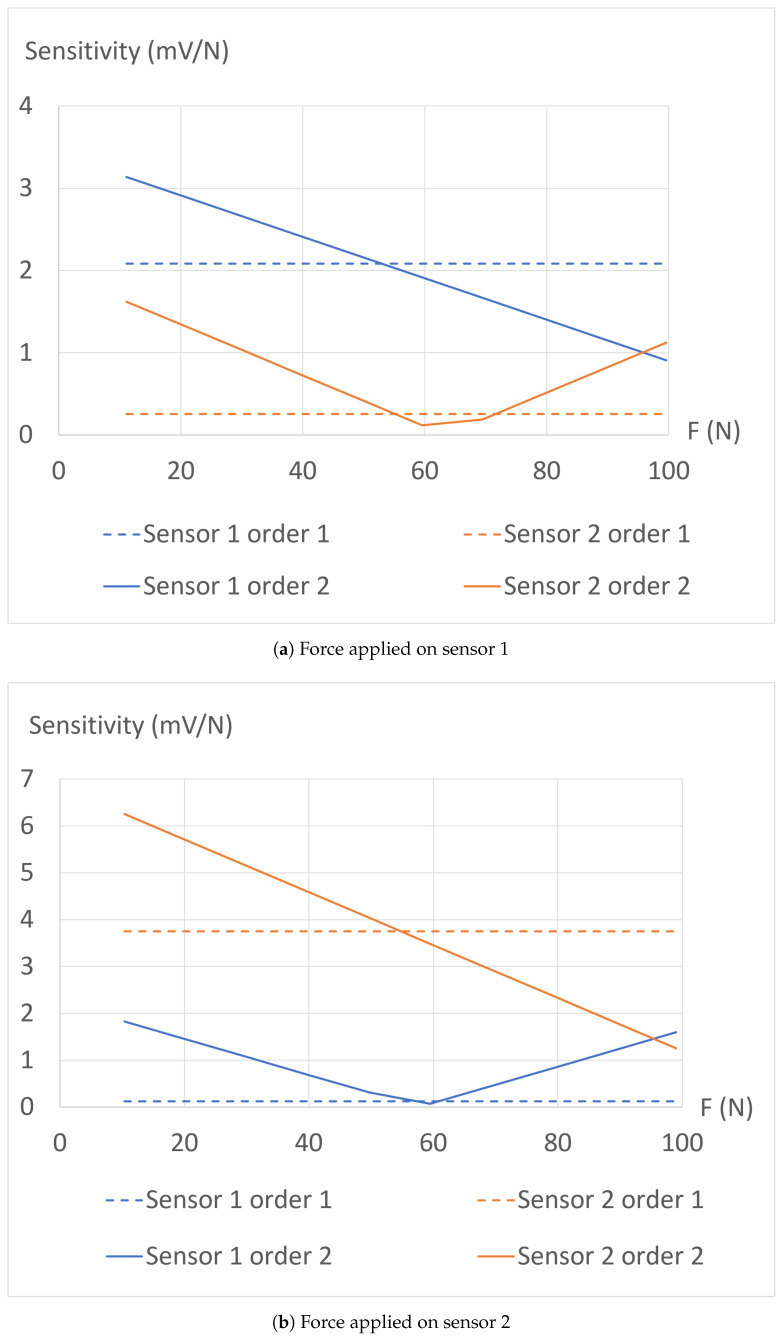
Sensitivity (absolute values) extracted from the first- and second-order regressions, for both sensors.

**Figure 10:**
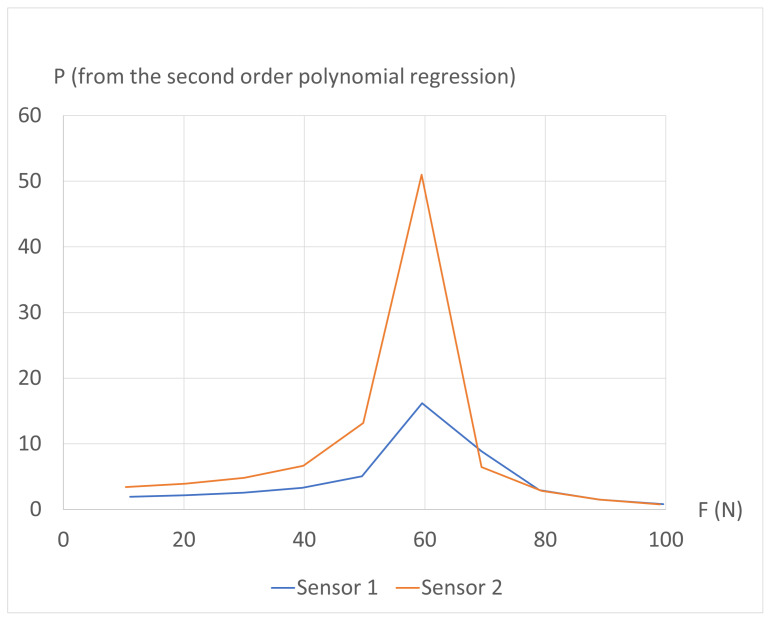
Evolution of the performance criterion with the applied force, based on the second-order regression.

Finally, the different absolute sensitivity values are plotted in Figure 9. These graphs provide a more comprehensive insight into the mechanical behavior of the structure during the experiment. The first-order regression highlights that the sprayed electrical ground effectively divides the structure into two sensing zones, although not perfectly. With the second-order regression, it becomes apparent that the sensitivity of the sensor under the mechanical constraint decreases, likely due to a reduction in the number of internal contacts created within the structure per Newton, as the force is being applied. Simultaneously, the sensitivity of the other sensor decreases due to the imperfect isolation between the two sensors, making their responses correlated. However, after surpassing a certain force threshold $F_{t}$ (around 60 N), another phenomenon takes precedence. Indeed, applying a force on the opposite side induces a Poisson effect, causing the sensor to slightly thicken, as predicted by the simulations presented earlier. Consequently, the structure experiences slight thickening or even delamination, leading to a longer average path for the electrical currents in this region, and therefore to a local increase in the electrical resistance. The combination of these effects causes the performance criterion *P* to initially rise until reaching a maximum at $F_{t}$, followed by a decrease down to values below 1, as depicted in Figure 10. This graph illustrates the limits of this first version of the 3D-printed piezoresistive force sensor array.

## 4. Discussion

The main objective of this preliminary study was to demonstrate the potential of this method intended to fabricate an array of independent force sensors within a single-material 3D-printed structure. The lattice structure allows for a soft and highly piezoresistive structure. It also increases the surface contact between the sprayed ground and the structure, in comparison with a flat surface, thus enhancing the electrical properties of this electrode. The subsequent phase of this project will involve printing a larger structure and subdividing it into more than two sensors, possibly creating the first version of a smart insole. However, the current structure is not yet optimized, exhibiting issues such as layer fatigue (resulting in delamination after a few cycles), non-linear sensitivity responses, and weak repeatability (according to the standard errors from Figure 7). Consequently, further investigation is imperative to identify more suitable materials and geometries to achieve enhanced performance. While this sensing principle holds promise for extension to more complex sensor structures with sprayed electrodes, the overarching goal of this method is to leverage additive manufacturing for tailored structures while circumventing some of its limitations, particularly in the context of electrode printing. This approach seeks to progressively integrate sensors into mechanical structures with improved performance, while using widespread printers and cheap materials. The fabrication process of the electrode is currently quite labor-intensive, as the sensors are sprayed one at a time, by hand. However, this could be drastically improved with pre-fabricated stencils able to cover a full batch of sensors. Furthermore, manual labor was necessary here for practical reasons, but it could completely be replaced by adapted spray-coating machines in a more industrial environment.

## 5. Conclusions

The technology introduced in this paper showcases the possibility to sense multiple forces with a single-material 3D-printed structure. We used a lattice structure made of a soft conductive polymer in combination with a spray-coated electrical ground dividing it into two distinct piezoresistive sensors. Experimental measurements have been effectively modeled using both first- and second-order polynomials. Each polynomial provides insights into different aspects of the sensors’ behavior. On the one hand, the first-order polynomial gives constant sensitivity for each sensor, underscoring the effectiveness of the electrical shielding. On the other hand, the second-order polynomial offers valuable information regarding the mechanical behavior and limits of the studied structure. As suggested in this paper, we believe that the selected lattice structure and material position our structure within the category of piezoresistive foams, from a macroscopic scale, because these foams can yield very high sensitivity due to local effects, as described in this paper. These structures can also exhibit low stiffness, making them suitable sensor solutions for soft machines and robots, but also in the medical field.

## Data Availability

Data are contained within the article.
